# Immunomodulatory Cross-Talk between Conjunctival Goblet Cells and Dendritic Cells

**DOI:** 10.1371/journal.pone.0120284

**Published:** 2015-03-20

**Authors:** Laura Contreras-Ruiz, Sharmila Masli

**Affiliations:** Department of Ophthalmology, Boston University School of Medicine, Boston, Massachusetts, United States of America; University of Birmingham, UNITED KINGDOM

## Abstract

Goblet cells are secretory epithelial cells of mucosal tissues that confer protection from environmental agents or pathogens via expression and secretion of soluble mucins. Loss of these cells is associated with several chronic inflammatory disorders of the mucosa. Although demonstrated to transfer antigens from the luminal surface to stromal cells in the intestinal mucosa, it is not known if goblet cells contribute to the regulation of an immune response. In this study we report that similar to intestinal and respiratory mucosal epithelia, mouse ocular surface epithelia predominantly express the TGF-ß2 isoform. Specifically, we demonstrate the ability of goblet cells to express TGF-ß2 and increase it in response to Toll-Like Receptor 4 mediated stimulus in cultures. Goblet cells not only express TGF-ß2, but are also able to activate it in a thrombospondin-1 (TSP-1) dependent manner via their cell surface receptor CD36. Furthermore, goblet cell derived soluble factors that possibly include TGF-ß2, alter dendritic cell (DC) phenotype to a tolerogenic type by downregulating DC expression of MHC class II and co-stimulatory molecules CD80, CD86 and CD40. Thus our study demonstrates goblet cells as a cellular source of active TGF-ß2 in ocular mucosa and implicates their immunomodulatory function in maintaining mucosal immune homeostasis.

## Introduction

The mucin secreting conjunctival epithelium forms a mucosal barrier between subepithelial immune cells and the environment. Similar to other mucosal surfaces the conjunctiva is endowed with its local lymphoid tissue, conjunctiva associated lymphoid tissue (CALT), comprised of cells capable of mounting innate and adaptive immune responses [1,2]. At steady state immunologic tolerance is induced against harmless antigens and commensal bacteria, while inflammatory immune response is mounted against pathogens to prevent infections [3]. Mechanisms underlying such homeostatic balance between tolerance and immunity at the ocular surface have not been fully explored.

By secreting mucins conjunctival epithelial cells, including goblet cells, are known to aid in the elimination of offending environmental agents [4]. The importance of goblet cells in particular in maintaining ocular surface homeostasis is well established [5]. Loss of these cells is a common feature in several inflammatory diseases of the ocular surface, including Stevens-Johnson syndrome, ocular mucous membrane pemphigoid, alkali burn, neutrophilic keratitis, graft-versus-host-disease, and Sjögren’s syndrome [6,7,8]. In addition to protecting the ocular surface via mucin secretion, goblet cells have been shown to contribute to the innate immune response by secreting mature IL-1ß via activation of the NLRP3 inflammasome [9]. However, unlike other mucosal surfaces contribution of conjunctival epithelial cells in priming the adaptive immune response has remained unaddressed.

The strategic location of goblet cells in the conjunctiva allows them direct contact with environmental agents and the conjunctival stroma that harbors dendritic cells (DCs). Dendritic cells in the conjunctiva are detected in organized follicles of CALT and diffusely distributed through the stroma along with intraepithelial lymphocytes [2,10,11]. Both CD11b+ and CD103+ subsets of CD11c+ dendritic cells are reported in murine conjunctiva and are known to contribute significantly to local immune responses [12]. Topically delivered soluble antigen on the ocular surface is detectable as associated with CD11c+ dendritic cells in the draining cervical lymph nodes [13]. Such dendritic cells capable of priming host adaptive immune responses are located in close proximity to mucin secreting goblet cells of the conjunctiva [11]. The structural location of goblet cells at the interface of the external environment and stromal immune cells makes them promising candidates for modulating the mucosal environment and as a consequence DC function and dependent immune responses. In this study, taking advantage of now feasible primary culture of murine conjunctival goblet cells, we investigate their potential role in modulating adaptive immune response. Although conjunctiva, as other mucosal surfaces, is a TGF-ß rich environment [14], it is not known if goblet cells serve as a cellular source of this immunomodulatory cytokine. Recently predominant expression of the TGF-ß2 isoform was reported in human conjunctival epithelial cells during chronic ocular surface inflammation [15]. In this study we evaluated if normal mouse conjunctiva, and specifically goblet cells, predominantly express this isoform and if its expression is modulated via toll-like receptor (TLR) stimulation. Moreover, it has been reported that due to an absence of the integrin binding RGD sequence in the LAP of the TGF-ß2 isoform, an integrin independent mechanism activates the latent form of TGF-ß2 [16]. Thrombospondin-1 (TSP-1), an extracellular matrix protein expressed by many ocular cell types, represents one such mechanism and has been shown to efficiently activate TGF-ß2 [17]. Therefore we determined if goblet cells activate their endogenous TGF-ß2, if any, in a TSP-1 dependent manner. In addition, to determine the immunomodulatory capacity of goblet cells we evaluated the effect of goblet cell derived soluble factors on dendritic cell phenotype.

The literature to date is limited in regard to goblet cells as sources of active TGF-ß, and to the best of our knowledge, no published report exists on the effect of goblet cells on adaptive immune response at the ocular surface. Our results demonstrate for the first time that conjunctival goblet cells can not only express and secrete an immunomodulatory cytokine, TGF-ß2, but can also activate it in a TSP-1 dependent manner via its receptor, CD36. Our results thus identify an as yet unknown immunomodulatory function of conjunctival goblet cells based on their ability to release soluble factors like TGF-ß2 and to modulate dendritic cell phenotype.

## Materials and Methods

### Mice

C57BL/6 (H-2b) mice, 4 to 12 weeks old, were purchased from Charles River Laboratories (Wilmington, MA). TSP-1 null mice (C57BL/6 background) were purchased from Jackson Laboratories (Bar Harbor, Maine). CD36−/− mice (C57BL/6 background) were obtained from the laboratory of Dr. M. Freeman (Massachusetts General Hospital, Harvard Medical School, Boston, MA). These mice were subsequently bred in-house in a pathogen-free facility at Boston University, Boston, MA. All animal experiments were conducted in accordance with institutional guidelines and the ARVO Statement for the Use of Animals in Ophthalmic and Vision Research.

### Ethics statement

The protocol (AN-15400) was approved by the Institutional Animal Care and Use Committee (IACUC) at Boston University School of Medicine.

### Isolation and culture of goblet cells

Goblet cells from mouse conjunctival pieces were grown in an organ culture, as described previously [18]. Briefly, conjunctival tissues were excised from 4- to 12-week-old male mice and placed in Hank’s balanced salt solution (Lonza, Walkersville, MD). Finely minced small tissue pieces were anchored onto a scratch at the bottom of a well in a 24-well plate. Sufficient RPMI-1640 medium supplemented with 10% heat-inactivated fetal bovine serum (Atlanta Biologicals, Lawrenceville, GA), 1 mM sodium pyruvate, 10 mM HEPES, 100 μg/mL penicillin/streptomycin, and 1X nonessential amino acid mixture (Lonza) was added to barely submerge the tissue. Explants were fed every other day with complete RPMI-1640 medium and grown under routine culture conditions of 5% CO_2_ at 37°C. Cells were grown from the tissue explant for 14 days to reach approximately 85% confluence; and then the explant was removed and discarded. To evaluate the effect of TLR stimulation on goblet cell function, cultured conjunctival goblet cells were maintained for 16 h in serum free medium, and then treated with Lipopolysaccharide (LPS) from *Pseudomonas aeruginosa* (Sigma-Aldrich, St. Louis, MO) for 24 h. A range of LPS concentrations (0.01 to 100 μg/mL) that did not significantly affect goblet cell viability, was tested and 1 μg/mL concentration was selected for subsequent experiments based on comparable results seen with concentrations up to 10 μg/mL.

### Immunostaining

For immunofluorescence microscopy of intact conjunctiva, eyes were enucleated with the lids intact, fixed in 4% formaldehyde in phosphate buffered saline (PBS) for 48 h, cryoprotected in 10–30% sucrose for 72 h, and embedded in OCT. Sections (7 μm) were placed on slides and kept at −20°C until use. For immunohistochemistry of cultured cells, primary cells were grown on glass coverslips and fixed in ice-cold methanol. Tissue sections and primary cells were blocked in PBS containing 4% goat or donkey serum, 0.3% Triton X-100, and 1% bovine serum albumin at RT for 1 h (all from Sigma-Aldrich), and incubated overnight at RT with the primary specific antibodies anti-TGF-ß1 (2 μg/ml), anti-TGF-ß2 (10 μg/ml) (both from R&D Systems, Minneapolis, MN), TSP-1 (2 μg/ml; Santa Cruz Biotech., Dallas, TX), and CD36 (2 μg/ml; Abcam, Cambridge, MA). After rinsing in PBS, AlexaFluor-conjugated secondary antibodies (Invitrogen, Eugene, OR) were used for 1 h at RT, cell nuclei counterstained with DAPI dye, and slides mounted and viewed under a FSX100 Olympus fluorescence microscope (Center Valley, PA).

### RT-PCR

Total RNA was isolated from conjunctivas harvested from WT (12 weeks, n = 5) and from cultured goblet cells using TRIzol Reagent (Life Technologies, Carlsbad, CA) according to the manufacturer’s instructions. cDNA was synthesized using the SuperScript VILO cDNA kit (Life Tech.). Real-time PCR was performed on the 7200 Real Time System (Applied Biosystems, Carlsbad, CA) using SYBR Green PCR Master Mix (Life Tech.) to determine relative quantitative expression levels of TGF-ß1, TGF-ß2, MHC class II, CD80, CD86 and CD40. TGF-ß1 primers (F-5’-TCAATACGTCAGACATTCGGGAAG-3’ and R-5’-GGTAACGCCAGGAATTGTTGCTAT-3’), TGF-ß2 primers (F-5’-AGGCGAGATTTGCAGGTATTGA-3’ and R-5’-GTAGGAGGGCAACAACATTAGCAG-3’), MHC class II primers (F-5’-AGGGCATTTCGTGTACCAGTT-3’ and R-5’-GTACTCCTCCCGGTTGTAGATGTA-3’), CD80 (F-5’-GAATTACCTGGCATCAATACGACA-3’ and R-5’-CTTAATGGTGTGGTTGCGAGTC-3’), CD86 (F-5’-TCACAAGAAGCCGAATCAG-3’ and R-5’-TGATAGTCTCTCTGTCAGCGTTAC-3’), CD40 (F-5’-CTTGTTGACAGCGGTCCATCTAGG-3’ and R-5’-TGGCCATCGTGGAGGTACTGTT-3’), and glyceraldehyde-3-phosphate dehydrogenase primers (F-5’-CGAGAATGGGAAGCTTGTCA-3’ and R-5’-AGACACCAGTAGACTCCACGACAT-3’) were used. Amplification reactions were set up in quadruplicates with the following thermal profile: 95°C for three minutes, 40 cycles at 95°C for 20 seconds, 55°C for 30 seconds, and 72°C for 40 seconds. To verify the specificity of the amplification reaction, a melting curve analysis was performed. Fluorescence signal generated at each cycle was analyzed using system software. The threshold cycle values were used to determine relative quantification of gene expression with glyceraldehyde-3-phosphate dehydrogenase as a reference gene.

### Flow cytometry

Cultured conjunctival goblet cells were stained with eFluor 780-conjugated Fixable Viability Dye (eBioscience, San Diego, CA). TGF-ß1 (R&D Systems), TGF-ß2 (R&D Systems), TSP-1 (Santa Cruz Biotech.), CD36 (eBioscience), and MHC class II (BD Bioscience, San Jose, CA) primary antibodies and AlexaFluor-conjugated secondary antibodies (Life Technologies) were used to assess surface staining. Their respective isotype-matched antibodies served as negative controls. Intracellular TGF-ß1, TGF-ß2 and TSP-1 were stained with the same antibodies but using an intracellular staining kit (eBioscience) as per the manufacturer’s instruction. Fluorescence-labeled cells were analyzed using BD LSRII Flow Cytometer (BD Bioscience). Further analysis of the data was performed using FlowJo v9.4.10 software (Tree Star, Inc., Ashland, OR).

### TGF-ß bioassay

The amount of active TGF-ß was measured using MFB-F11 cells, a fibroblast cell line derived from TGF-ß–knockout mice and stably transfected with the SBE-SEAP reporter [19]. Briefly, MFB-F11 cells were seeded in complete DMEM at a concentration of 3 × 10^4^ cells into flat-bottomed 96-well plates. After overnight incubation, cells were washed twice with PBS and incubated in 50 μl serum-free DMEM for 2 h before culture supernatant from goblet cells were added. Dilutions of TGF-ß2 (R&D Systems) were used as a standard. For measurement of total TGF-ß, supernatants were acidified with 1 N HCl for 10 minutes and then neutralized with 1 N NaOH right before application. Active TGF-ß was measured from untreated supernatants. To assess active TGF-ß2, supernatants were incubated with 1 μg/ml of TGF-ß2 neutralizing antibody (R&D Systems) for 1 h at room temperature. Isotype IgG antibody (Abcam) was used as control. After 24 h, culture supernatants were tested for SEAP activity using Great EscAPe SEAP Reporter system 3 (Clontech, Mountain View, CA) according to the manufacturer’s instructions, and absorbance was measured with a Synergy H1 microplate reader (Biotek, Winooski, VT). Samples were measured in triplicate.

### Transwell co-culture

Goblet cells were grown for 10 days on 6.5-mm transwell polyester membranes with 0.4 μm pores (Costar, Corning, NY) in complete RPMI-1640. Medium was refreshed every other day. Hematoxylin and eosin (H&E) stained 8-μm sections of paraffin embedded membranes were examined to confirm multilayered growth of cells. Bone marrow derived dendritic cells (BMDCs) were generated by culturing 1 x 10^6^ cells/ml with GM-CSF (20 ng/ml) for 7 days. For the co-culture, BMDCs (1 x 10^6^ /well) were added basolaterally and the culture plate was incubated at 37°C for 24 h. Harvested DCs at the end of this culture period were stained for surface markers and analyzed by flow cytometry or processed to harvest RNA as described above.

### Statistical analysis

Student’s t-test was used to determine significant differences between mean values of experimental and control groups. Error bars in Figures represent ± standard error of the mean (SEM). P < 0.05 was considered statistically significant.

## Results

### TGF-ß2 is the predominant isoform in conjunctival epithelium

**Fig 1:**
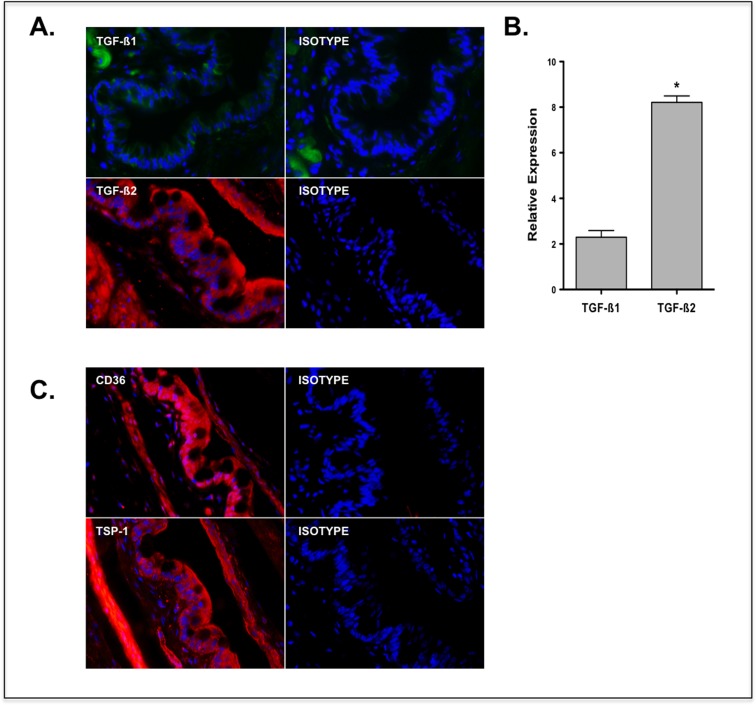
Conjunctival epithelium predominantly expresses TGF-ß2 and molecules associated with its activation, TSP-1 and CD36. Expression of TGF-ß1 and -ß2 isoforms in conjunctiva was assessed using (a) immunofluorescence and (b) real time PCR. Both isoforms were detected in conjunctival epithelium, including goblet cell rich areas, with a significantly higher expression of TGF-ß2. Results are presented as relative expression to that of the housekeeping gene GAPDH. (c) Immunostaining for TSP-1 and its receptor CD36 was detected in WT mouse conjunctival epithelium. Nuclei were counterstained with DAPI (blue). Magnification = x40. (*P < 0.05 compared to TGF-ß1)

Both TGF-ß1 and TGF-ß2 isoforms are expressed in the human ocular surface. Similar to other mucosal surfaces, such as intestinal or airway mucosa, TGF-ß2 is the predominant isoform detectable in human conjunctiva [20]. To evaluate the presence of TGF-ß isoforms in a normal mouse ocular surface mucosa, we examined conjunctival expression of TGF-ß1 and TGF-ß2 isoforms using immunofluorescence staining followed by fluorescence microscopy. As shown in Fig. 1A, immunostaining for both TGF-ß1 and -ß2 isoforms was detected in conjunctival epithelium, with an obvious predominance of TGF-ß2 consistent with its elevated message level as detected by real-time PCR (Fig. 1B).

Activation of latent TGF-ß at most mucosal surfaces is largely attributed to integrins. However, integrins fail to activate latent TGF-ß2 [16], while it is efficiently activated by TSP-1 [17]. At the cell surface, TSP-1 is reported to achieve activation of TGF-ß via binding to its receptor CD36 [21]. To determine the potential use of a TSP-1 mediated TGF-ß-activation mechanism by conjunctival epithelial cells we evaluated the expression of TSP-1 and its receptor CD36 in the conjunctiva. As shown in Fig. 1C, significant TSP-1 as well as CD36 immunostaining was detected in the epithelial layer of the mouse conjunctiva.

These results demonstrate that, as in humans, TGF-ß2 is the predominant isoform expressed in mouse conjunctival epithelium and that expression of molecules associated with its efficient activation such as TSP-1 and CD36 are also expressed in the epithelial layer of the tissue.

### Conjunctival goblet cells predominantly express TGF-ß2 isoform

To determine whether conjunctival goblet cells specifically produce TGF-ß, we used a primary culture of goblet cells derived from conjunctiva tissue of WT mice. We have previously described that such cultured goblet cells isolated from mouse conjunctiva retain goblet cell specific markers like MUC5AC and also secretory function [18].

**Fig 2:**
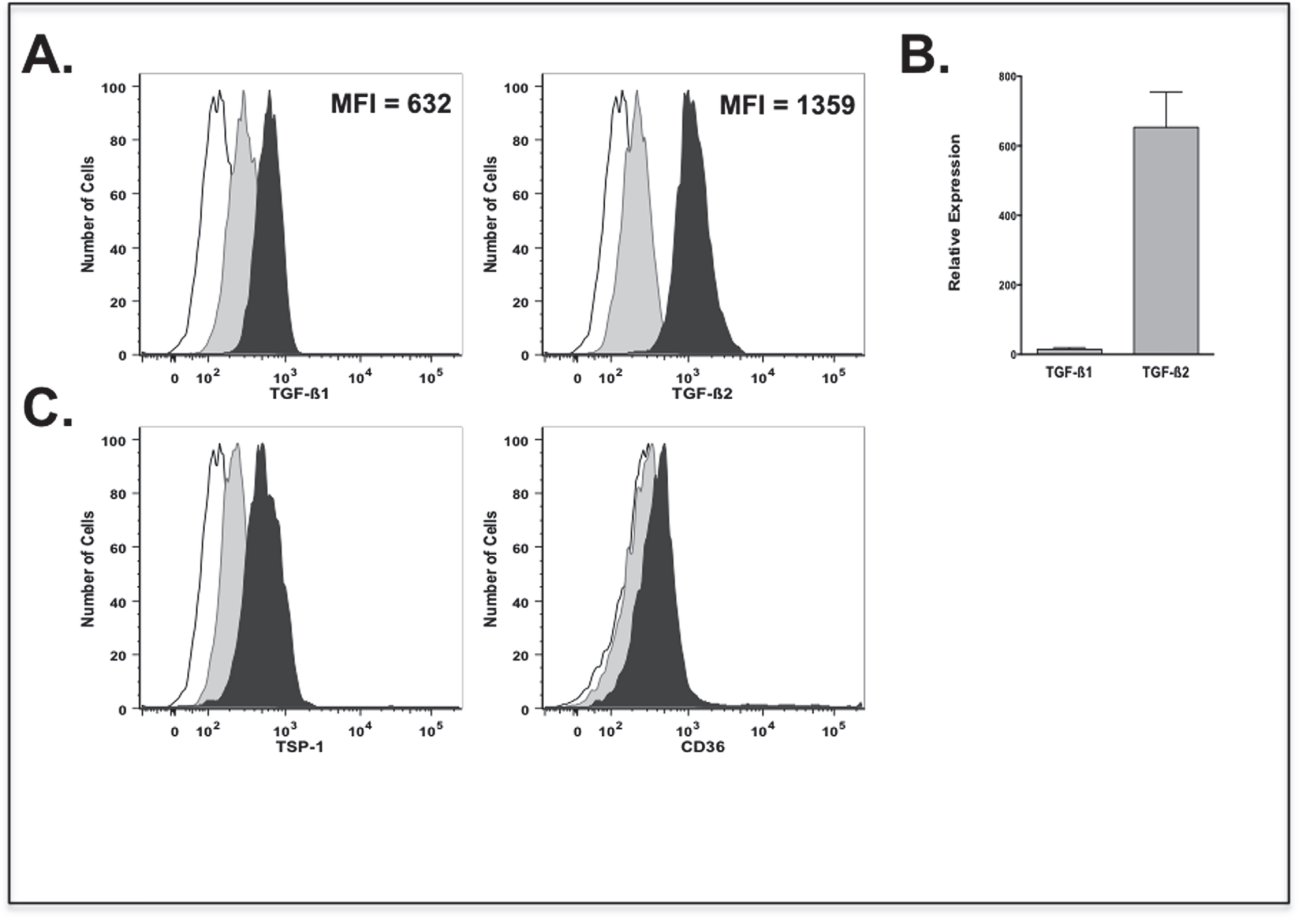
Cultured conjunctival goblet cells express TGF-ß2, TSP-1 and CD36. (a) The expression of TGF-ß1 and TGF-ß2 isoforms in primary cultures of conjunctival goblet cells was assessed using flow cytometry. Representative histograms from 3 independent experiments: unstained cells (empty histogram), isotype controls (grey histogram), and cultured goblet cells (solid histogram). The mean fluorescence Intensity (MFI) of TGF-ß2 staining was significantly increased as compared to TGF-ß1. (b) Expression of TGF-ß1 and -ß2 isoforms was evaluated in cultured goblet cells using real time PCR. Results are presented as relative expression to that of the housekeeping gene GAPDH. (*P < 0.05 compared to TGF-ß1). (c) Immunostaining for TSP-1 and its receptor CD36 was detected in cultured goblet cells using flow cytometry.

Flow cytometric analysis of cultured goblet cells indicated expression of both TGF-ß1 and TGF-ß2 isoforms as shown in Fig. 2A. However, analysis of the mean fluorescence intensity revealed significantly increased expression of TGF-ß2 as compared to that of TGF-ß1. Consistently, real-time PCR analysis indicated predominant TGF-ß2-specific message (Fig. 2B). These results are consistent with the conjunctival tissue expression of TGF-ß2. Moreover immunostaining for both TSP-1 and CD36 was detectable in cultured goblet cells as shown in Fig. 2C. Together these results clearly demonstrate that goblet cells not only serve as a source of TGF-ß2 in the ocular mucosa but also are also equipped with molecules essential for generating its biologically active form.

### Conjunctival goblet cells up-regulate the expression, secretion and activation of TGF-ß2 in response to LPS stimulation

**Fig 3:**
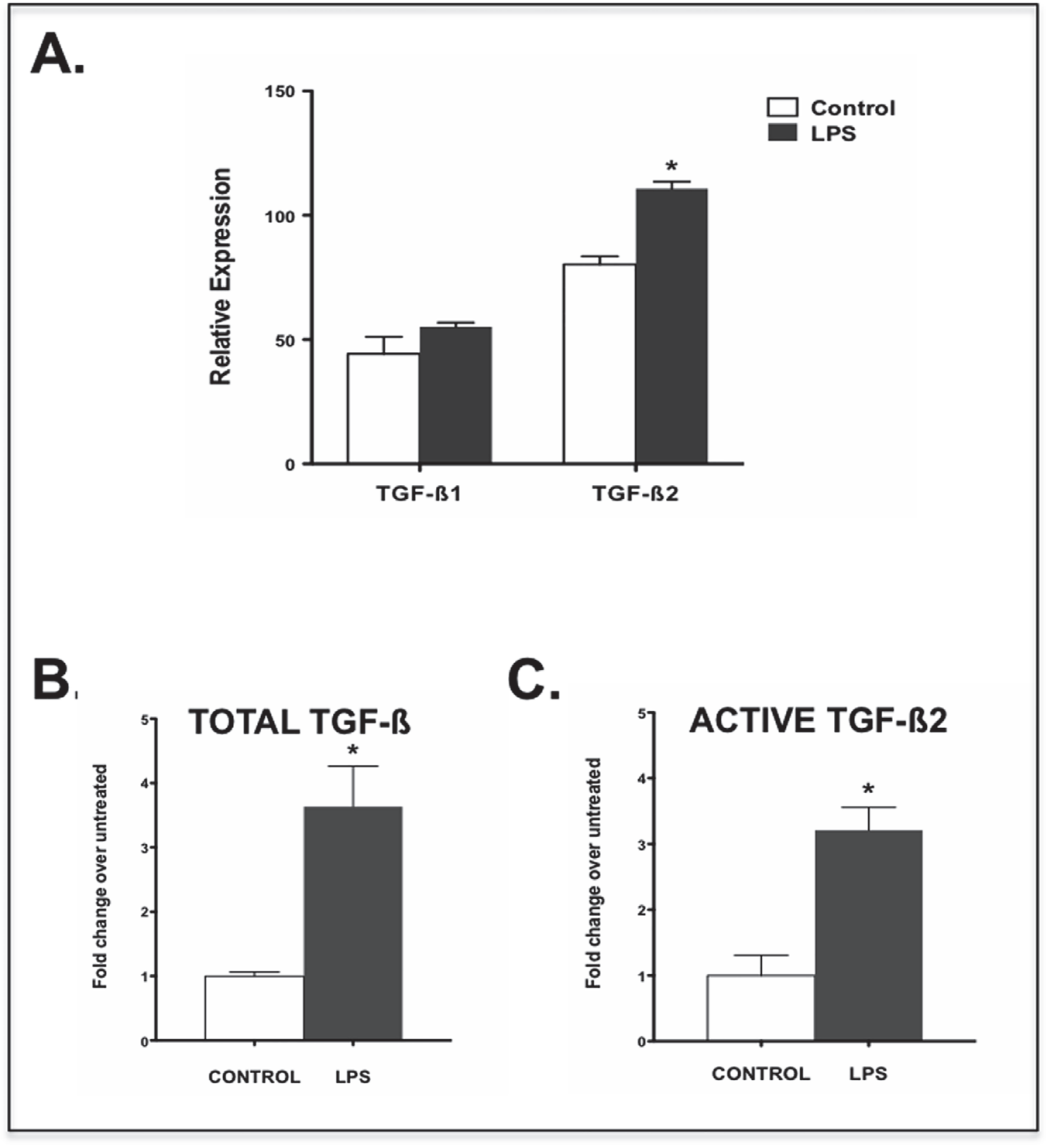
Stimulation of cultured goblet cells with LPS induces increased expression, secretion and activation of TGF-ß2. (a) Expression of TGF-ß1 and -ß2 in LPS-exposed goblet cells was determined using real time PCR. Results are presented as relative expression to that of the housekeeping gene GAPDH. (b) Total TGF-ß and (c) active TGF-ß2 secreted by LPS-exposed goblet cells were measured using MFB-F11 reporter cells as described in methods. Changes in the levels relative to those secreted by untreated cells are presented. (*P < 0.05).

Conjunctival goblet cells are known to contribute to the mucosal barrier via their mucin secretion [4]. However, their ability to express TGF-ß2 and its activation related molecules suggests its ability to provide active TGF-ß2 in the tissue microenvironment. Considering the exposure of ocular mucosa to normal microflora we assessed whether TLR mediated stimulation of goblet cells may modulate their ability to express TGF-ß2. We exposed WT primary goblet cell cultures to LPS (1 μg/mL), a TLR4 ligand, for 24h and assessed their expression of TGF-ß1 and TGF-ß2 isoforms in a real-time PCR assay. As shown in Fig. 3A, while TGF-ß2 expression in goblet cells exposed to LPS was significantly increased as compared to that in untreated cells, no change in TGF-ß1 expression was detectable. We next determined whether such increase in TGF-ß2 message level in conjunctival goblet cells corresponds to an increased **s**ecretion of total TGF-ß. To quantitate secreted TGF-ß we determined total TGF-ß in serum-free culture supernatants collected from LPS-exposed goblet cells using the TGF-ß-SEAP reporter cell line (MFB-F11) [19]. Acid treatment of supernatants followed by its neutralization allowed assessment of total TGF-ß. As shown in Fig. 3B, significantly increased TGF-ß was detected in culture supernatants of LPS-stimulated conjunctival goblet cells as compared to untreated control cells.

Detection of TGF-ß2-activation related molecules like TSP-1 and CD36 by cultured goblet cells led us to determine relative levels of the bioactive TGF-ß2 isoform in culture supernatants of goblet cells cultured in serum-free medium. This was achieved by assessing the effect of selective blockade of the isoform in culture supernatants using specific neutralizing antibody. Prior to this study the specificity of the anti-TGF-ß2 antibody was determined by abrogating bioactivity of rTGF-ß2 in the same assay. While a non-specific isotype control antibody did not block rTGF-ß2 bioactivity, the specific antibody blocked more than 90% of this bioactivity (data not shown). After incubation of culture supernatants collected from untreated or LPS-stimulated cells with anti-TGF-ß2 antibodies, the concentration of TGF-ß2 was deduced as a difference from total active TGF-ß. Results shown in Fig. 3C represent bioactivity attributable to the TGF-ß2 isoform in culture supernatants derived from untreated or LPS-stimulated conjunctival goblet cells. A 3-fold increase in active TGF-ß2 was detectable in supernatants from LPS stimulated goblet cells compared to that from untreated control cells.

Together, these results demonstrate that conjunctival goblet cells, not only up-regulate the expression and secretion of TGF-ß2, but also have the ability to activate this isoform in response to a TLR4 mediated stimulus. The proximity of conjunctival goblet cells to antigen presenting cells in combination with an ability to release the biologically active form of TGF-ß2 in the tissue environment strongly implicates an immunomodulatory role of goblet cells in the ocular mucosa.

### Colocalization of TSP-1, CD36 and TGF-ß2 in the membrane of conjunctival goblet cells

**Fig 4:**
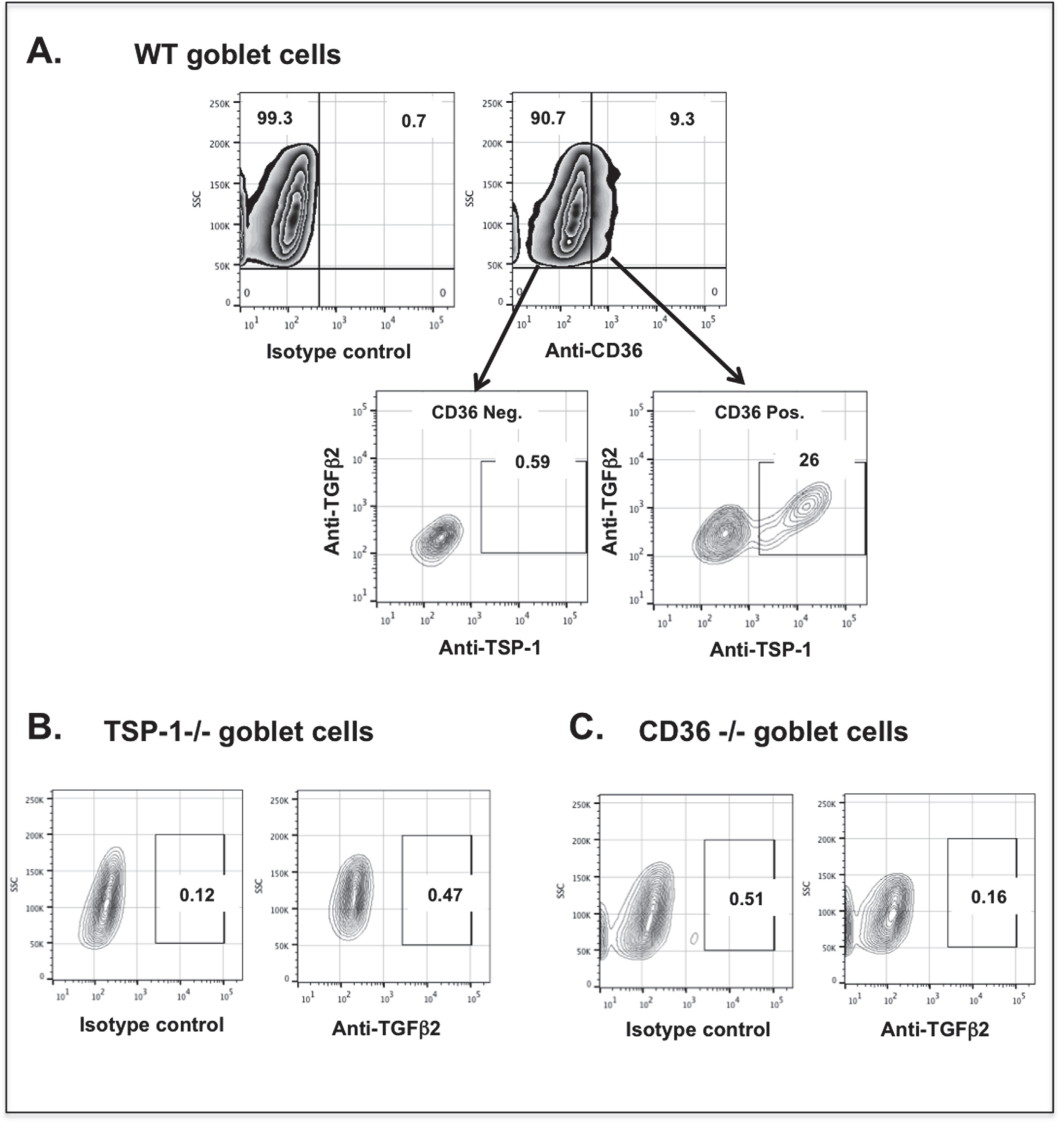
Thrombospondin-1, CD36 and TGF-ß2 colocalize on the goblet cell surface. Flow cytometry analysis of the surface staining of CD36, TSP-1 and TGF-ß2 on cultured goblet cells derived from WT (a), TSP-1 −/− (b) or CD36 −/− (c) mice. Membrane CD36+, TSP-1+ and TGF-ß2+ goblet cells were detected in WT cultures, while no membrane bound TGF-ß2 was detectable in goblet cells derived from either TSP-1 −/− or CD36 −/− mice. Data are representative of 2 independent experiments.

With the conclusive identification of goblet cells as a source of active TGF-ß2, we next moved on to assess the mechanisms involved in this activation. Binding of TSP-1 to its receptor CD36 is known to facilitate the activation of latent TGF-ß in alveolar macrophages [21]. To determine if CD36 detected on conjunctival goblet cell surface binds TSP-1 and latent TGF-ß2 complex, we detected membrane bound molecules by flow cytometric analysis of goblet cells stained for CD36, TSP-1 and TGF-ß2. As shown in Fig. 4A, analysis of WT goblet cells identified 9.3% cells stained for CD36. Of these, 26% of cells stained positive for both TSP-1 and TGF-ß2, while both these molecules were undetectable on CD36 negative goblet cells. To confirm colocalization of the three molecules in goblet cell membranes, we analyzed similarly stained goblet cells derived from TSP-1 or CD36 deficient mice. As shown in Fig. 4B and C, no membrane bound TGF-ß2 was detectable in goblet cells derived from either strain. Together these results indicate that TSP-1, CD36, and TGF-ß2 colocalize on the membrane of conjunctival goblet cells further confirming their ability to utilize TSP-1 to activate their TGF-ß2.

### Conjunctival goblet cells depend on TSP-1 to activate TGF-ß2

**Fig 5:**
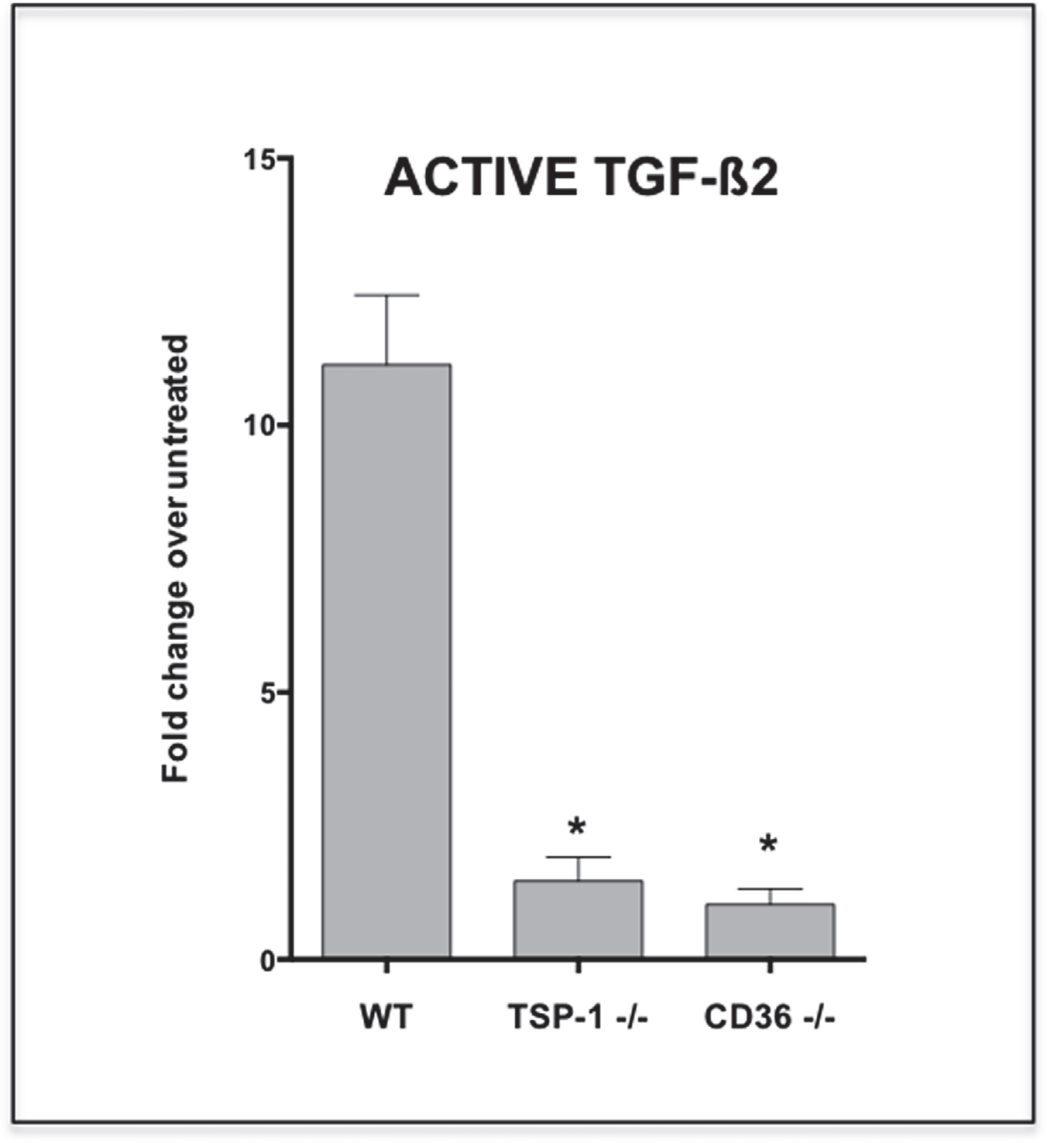
Goblet cells deficient in TSP-1 or CD36 fail to release active TGF-ß2. Activation of TGF-ß2 by LPS-exposed WT, TSP-1 deficient or CD36 −/− cultured conjunctival goblet cells was assessed using MFB-F11 reporter cell line. Unlike WT, both TSP-1 and CD36 deficient goblet cells failed to increase their active TGF-ß2 in response to LPS exposure in culture. Changes in the levels relative to the untreated goblet cells are presented. (*P < 0.05 compared to WT)

To determine if the membrane colocalization of TSP-1, CD36, and TGF-ß2 is essential for the TGF-ß2 activation by goblet cells, we evaluated the ability of TSP-1 or CD36 deficient goblet cells to activate their TGF-ß2. We first confirmed by real-time PCR that all the three tested goblet cells (WT, TSP-1−/− and CD36−/−) express comparable levels of TGF-ß2 message upon their LPS exposure (WT: 1.6 ± 0.1; TSP-1−/−: 1.4 ± 0.1; CD36−/− 2.1 ± 0.1 fold increase over untreated). We then tested active TGF-ß2 content of their supernatants using MFB-F11 reporter cells. Unlike WT goblet cells that responded to LPS-exposure with over 10-fold increase in secreted active TGF-ß2, both TSP-1 and CD36 deficient goblet cells failed to increase their active TGF-ß2 secretion in response to LPS exposure in culture (Fig. 5). Thus our results indicate that conjunctival goblet cells indeed depend on TSP-1 to activate their own TGF-ß2.

### Conjunctival goblet cells modulate dendritic cell phenotype

**Fig 6:**
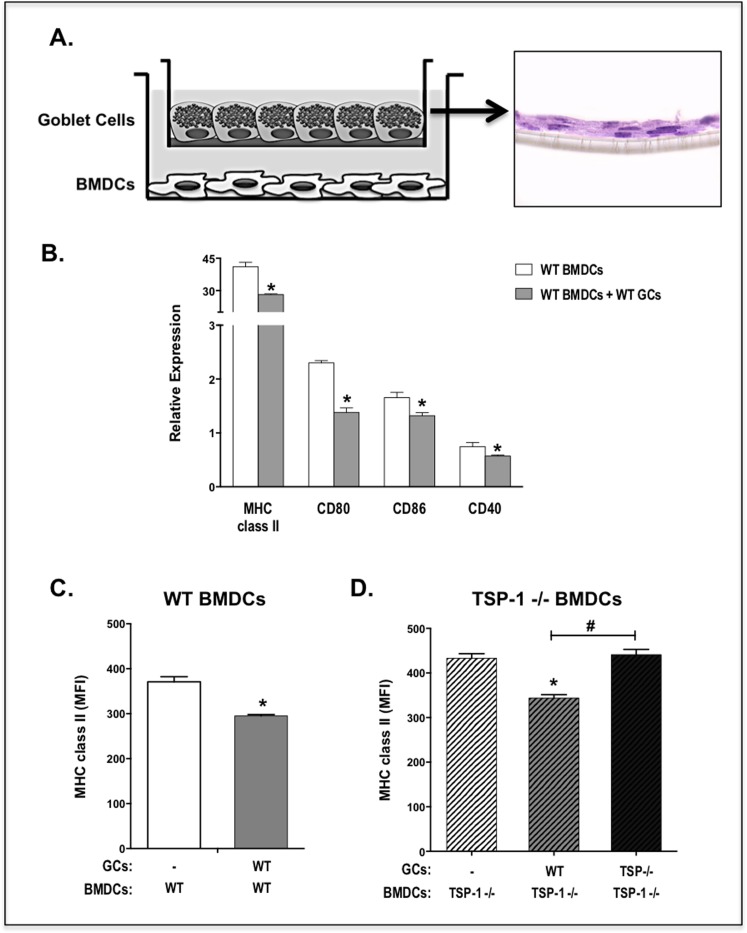
Goblet cell derived TGF-ß2 contributes to the tolerogenic phenotype of dendritic cells. (a) Cultured WT goblet cells were grown to confluence on transwell polyester membranes. Multilayered growth was confirmed by H&E staining. WT BMDCs were cultured in the lower chamber. (b) After 24 h of co-culture, BMDCs were harvested and the expression of MHC class II, CD80, CD86 and CD40 was analyzed by RT-PCR. Surface MHC class II staining was assessed by flow cytometry on WT (c) or TSP-1 deficient (d) BMDCs co-cultured with indicated goblet cells. Results are presented as relative expression to that of the housekeeping gene GAPDH, and as mean fluorescence intensity (MFI). (*P < 0.05).

One of the immunoregulatory activities of TGF-ß includes signaling in DCs to maintain their immature and tolerogenic state characterized by low expression of MHC class II and co-stimulatory molecules [22,23]. Considering the ability of goblet cells to express, secrete and activate TGF-ß2 we evaluated their ability to modulate DC phenotype. We first cultured WT goblet cells in transwell inserts and confirmed their multilayered growth on polyester membranes by examining H&E stained sections of these membranes (Fig. 6A). We then cultured WT BMDCs in the lower chambers of transwell plates to prevent their direct contact with goblet cells for a period of 24 h before harvesting their RNA. Real-time PCR analysis of this RNA was performed to detect MHC class II, CD80, CD86 and CD40 specific message levels. As shown in Fig. 6B, a significantly reduced expression of all these markers was detectable in BMDCs co-cultured with goblet cells compared to control BMDCs cultured alone. Flow cytometric assessment of surface MHC class II staining in BMDCs further confirmed reduced expression of MHC class II in BMDCs co-cultured with goblet cells (Fig. 6C).

To specifically determine the relevance of goblet cell derived active TGF-ß2 and to eliminate any potential influence of DC-derived TGF-ß2, in the regulation of DC phenotype in our experiments, we used TSP-1 deficient DC that are incapable of activating TGF-ß2. In these cultures only GCs serve as a source of active TGF-ß2. As shown in Fig. 6C, a significantly reduced expression of MHC class II was detectable in TSP-1 deficient BMDCs co-cultured with WT goblet cells compared to control BMDCs cultured alone. However, this effect on MHC class II expression on was lost if goblet cells were deficient in TSP-1.

Together these results suggest that goblet cell derived TGF-ß2 that is activated in a TSP-1 dependent manner modulates DC phenotype towards an immature or tolerogenic state. In fact, MHC class II expression in TSP-1−/− BMDCs was significantly increased compared to WT BMDCs cultured alone. Therefore the additional possibility of a release of any pro-inflammatory factor(s) by TSP-1 deficient goblet cells cannot be ruled out and requires further investigations.

## Discussion

**Fig 7:**
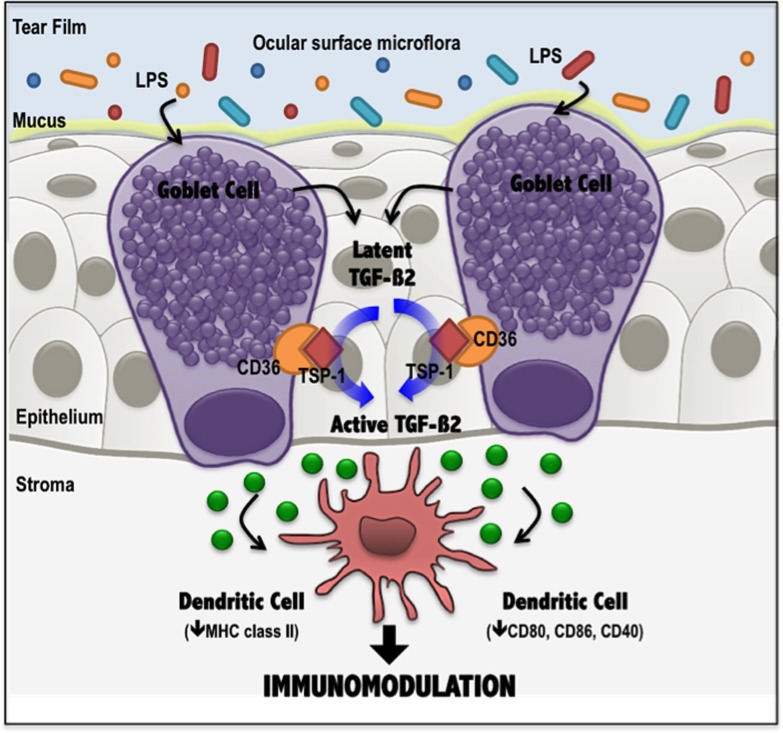
Immunomodulatory function of conjunctival goblet cells in ocular surface mucosa. In response to an LPS-mediated stimulus, conjunctival goblet cells increase expression of TGF-ß2, and activate it in a TSP-1 dependent manner via a TSP-1 receptor, CD36. Such goblet cell derived active TGF-ß2 modulates the phenotype of surrounding dendritic cells toward an immature or tolerogenic state, decreasing the expression of activation markers, such as MHC class II, CD80, CD86 and CD40.

In this study we report a first investigation of goblet cells as a source of the active isoform TGF-ß2 which predominates in most mucosal epithelia. While epithelial cells in the conjunctiva express the TGF-ß2 isoform constitutively, goblet cells were found to increase this expression in response to a TLR4-mediated stimulus. In addition to their well-known mucin secreting function, our results demonstrate the ability of goblet cells to not only express TGF-ß2 but also activate it in a TSP-1 dependent manner. Such goblet cell derived active TGF-ß2 also modulates dendritic cell phenotype towards a tolerogenic type. Thus our study demonstrates conjunctival goblet cells as a cellular source of TGF-ß2 in ocular mucosa and implicates their immunomodulatory function in maintaining immune homeostasis at the ocular surface that is persistently exposed to commensal flora and the external environment (Fig. 7).

Although several cell types at mucosal surfaces have been reported to serve as a source of TGF-ß, specifically predominant expression of the TGF-ß2 isoform was reported in human airway and intestinal epithelial cells [24,25]. Our results in murine ocular surface epithelia are consistent with these studies. Moreover, increased expression of this isoform was reported in mucosal epithelia of human patients with inflammatory conditions like asthma, dry eye and vernal keratoconjunctivitis where the mucosal epithelium is damaged [15,20,26,27]. In an *in vitro* study bronchial epithelial-derived TGF-ß2 was reported to contribute to airway repair under normal conditions as well as in response to epithelial injury by modulating the extracellular matrix structure suggesting that tight regulation of TGF-ß2 is necessary for extracellular matrix homeostasis [28]. While several studies report significant changes in goblet cell numbers or their expression of host defense molecules during mucosal inflammatory conditions [29,30], no studies have addressed their ability to express TGF-ß. Our study not only demonstrates the ability of goblet cells to express and secrete active TGF-ß2 but also shows their ability to modulate this expression in response to a TLR4 mediated stimulus.

There is evidence that TLR4 plays an important role in intestinal homeostasis and protection from injury to intestinal mucosa in inflammatory bowel disease [31,32]. In these studies TLR4 deficient mice are reported to develop severe DSS-induced colitis suggesting that TLR4 stimulation in intestinal mucosa by gut microflora results in secretion of factors that promote epithelial restitution after injury. The protective function of TLR4 in the mucosal tissue and TGF-ß2-mediated mucosal tissue repair together suggest that TLR4-induced goblet cell-derived TGF-ß2 may serve as a protective factor in mucosal tissue in steady state. Furthermore, findings from the intestinal mucosa emphasize that dysregulation of a protective response can lead to a chronic inflammatory condition. The biological activity of the predominant TGF-ß2 isoform in mucosal epithelia needs an integrin independent mechanism like TSP-1. Therefore TSP-1 deficiency in mice can be expected to effectively dysregulate the protective function of TGF-ß2 at mucosal surfaces. In line with this possibility TSP-1 deficient mice spontaneously develop chronic ocular surface inflammation that progresses with age [13,33]. Similarly it is reported that both bleomycin induced injury to lung epithelium or DSS-induced injury to intestinal epithelium in TSP-1 deficient mice results in exacerbation of chronic inflammatory condition [34,35]. Together these studies strongly support the contention that TSP-1 deficiency in mucosal tissues may dysregulate the protection conferred by TGF-ß2 against injury at mucosal surfaces. This possibility is also supported by the observations made in human subjects. In two separate studies reduced TSP-1 expression in either colonic or ocular mucosal epithelia was associated with chronic inflammation as seen in inflammatory bowel disease and dry eye respectively [36,37].

Our findings also lend themselves to a possibility that ocular surface microflora is likely to play a role in conjunctival homeostasis via ligation of TLR4 on goblet cells releasing TGF-ß2 while relying on TSP-1 to activate it. This would mean that the absence of TLR4 ligation by ocular surface commensal flora may result in ocular surface inflammation. Indeed in a recent study C57BL/6 mice depleted of commensals with broad-spectrum antibiotics were reported to develop a severe form of dessicating-stress-induced ocular surface disease [38]. Interestingly, at the highest LPS concentration (100 mg/ml) tested in our study we noted significantly downregulated expression of TGF-ß2 in goblet cells (data not shown). Therefore it is possible that altered tear quality in dry eye favors growth of TLR4-engaging bacteria at the ocular surface resulting in signals equivalent to high LPS concentration in our study thereby disrupting the immunomodulatory function of goblet cells and promoting inflammation. In another study a favorable therapeutic response to cyclosporine in patients with ocular surface inflammation involved an increase in conjunctival goblet cell numbers accompanied with an increase in TGF-ß2 expression [39]. These studies further support an important role of the TGF-ß2 isoform in maintaining homeostasis in the ocular mucosa.

The maintenance of immune homeostasis at mucosal surfaces is largely attributed to resident antigen presenting cells such as DCs that are abundant in organized lymphoid organs and lamina propria. Several DC subsets have been identified in mucosal tissues. In particular, a TGF-ß2 expressing intestinal CD103+ subset is known to induce Foxp3+ regulatory T cells based on tolerogenic conditioning provided by the tissue environment [40,41]. Likewise functionally divergent CD103+ and CD11b+ subsets of CD11c+ DCs are detected in the ocular mucosa [12]. These conjunctival CD11c+ DCs carry topically applied antigens to the draining cervical lymph nodes [13]. Maturation of DCs is considered a fundamental checkpoint in the initiation and shaping of the immune response. In the present study, we demonstrate that conjunctival goblet cells are actively engaged in regulating DC maturation. Conjunctival goblet cells under homeostatic conditions provide an active TGF-ß2 containing microenvironment in a TSP-1 dependent manner. Such an environment in the tissue then promotes a tolerogenic DC phenotype by lowering the expression of their maturation markers such as MHC class II and co-stimulatory molecules. Indeed DCs exposed to TGF-ß2, *in vitro*, induce TGF-ß1 secreting functional CD4+CD25+Foxp3+ Tregs (Mir et al., manuscript submitted). Our study suggests that ocular surface microflora may aid in maintainance of the tolerogenic tissue environment thereby influencing the adaptive immune response. These findings underscore an immunomodulatory role of conjunctival goblet cells. Although participation of goblet cells in innate response has been previously described [9], this is, to our knowledge, a first report describing the potential of conjunctival goblet cells to modulate an adaptive immune response. Therefore, possibly the loss of these cells in inflammatory diseases contributes to a loss of immune homeostasis and consequential chronic inflammation.

We conclude that conjunctival goblet cells can not only express and secrete the immunomodulatory cytokine TGF-ß2 but can also activate it in a TSP-1 dependent manner. Moreover, our results identify an as yet unknown immunomodulatory function of conjunctival goblet cells based on their ability to release soluble factors like TGF-ß2 and to modulate dendritic cell phenotype. This study also suggests that by virtue of its ability to efficiently activate TGF-ß2, TSP-1 is likely to be an important contributor to regulatory mucosal immunity beyond ocular mucosa.
